# Supplementary material for: Correlation between pre-operative VE-cadherin and DLL4 and the maturation after primary arteriovenous fistula in uremic patients
Source: PeerJ. 2024 Nov 20;12:e18356. doi: 10.7717/peerj.18356 (PMC11585290; doi:10.7717/peerj.18356)
Supplement: Supplemental Information 7 [file peerj-12-18356-s007.pdf]

# Confidence Intervals for One Proportion

## Numeric Results for Two-Sided Confidence Intervals for One Proportion
## Confidence Interval Formula: Exact (Clopper-Pearson)

| Confidence Level | Sample Size (N) | Target Width | Actual Width | Proportion (P) | Lower Limit | Upper Limit | Width if P = 0.5 |
|---|---|---|---|---|---|---|---|
| 0.950 | 44 | 0.200 | 0.198 | 0.900 | 0.772 | 0.970 | 0.309 |

## References

Fleiss, J. L., Levin, B., Paik, M.C. 2003. Statistical Methods for Rates and Proportions. Third Edition. John Wiley & Sons. New York.

Newcombe, R. G. 1998. 'Two-Sided Confidence Intervals for the Single Proportion: Comparison of Seven Methods.' Statistics in Medicine, 17, pp. 857-872.


## Report Definitions
Confidence level is the proportion of confidence intervals (constructed with this same confidence level, sample size, etc.) that would contain the population proportion.
N is the size of the sample drawn from the population.
Width is the distance from the lower limit to the upper limit.
Target Width is the value of the width that is entered into the procedure.
Actual Width is the value of the width that is obtained from the procedure.
Proportion (P) is the assumed sample proportion.
Lower Limit is the lower limit of the confidence interval.
Upper Limit is the upper limit of the confidence interval.
Width if P = 0.5 is the maximum width for a confidence interval with sample size N.

## Summary Statements
A sample size of 44 produces a two-sided 95% confidence interval with a width equal to 0.198 when the sample proportion is 0.900.

## Dropout-Inflated Sample Size

| Dropout Rate | Sample Size N | Dropout-Inflated Enrollment Sample Size N' | Expected Number of Dropouts D |
|---|---|---|---|
| 20% | 44 | 55 | 11 |

## Definitions
Dropout Rate (DR) is the percentage of subjects (or items) that are expected to be lost at random during the course of the study and for whom no response data will be collected (i.e. will be treated as "missing").
N is the evaluable sample size at which the confidence interval is computed. If N subjects are evaluated out of the N' subjects that are enrolled in the study, the design will achieve the stated confidence interval.
N' is the total number of subjects that should be enrolled in the study in order to end up with N evaluable subjects, based on the assumed dropout rate. After solving for N, N' is calculated by inflating N using the formula N' = N / (1 - DR), with N' always rounded up. (See Julious, S.A. (2010) pages 52-53, or Chow, S.C., Shao, J., and Wang, H. (2008) pages 39-40.)
D is the expected number of dropouts. D = N' - N.

## Confidence Intervals for One Proportion

### Procedure Input Settings

**Autosaved Template File**
C:\Users\lenovo\Documents\PASS 15\Procedure Templates\Autosave\Confidence Intervals for One Proportion - Autosaved 2022_5_25-7_57_14.t16

**Design Tab**

| | |
|---|---|
| Solve For: | Sample Size |
| Confidence Interval Formula: | Exact (Clopper-Pearson) |
| Interval Type: | Two-Sided |
| Confidence Level (1 - Alpha): | 0.95 |
| Confidence Interval Width (Two-Sided): | 0.2 |
| P (Proportion): | 0.9 |
